# The iBuddy experience: A digital simulation-based approach to enhance secondary school students’ privacy awareness

**DOI:** 10.1007/s11423-023-10309-x

**Published:** 2023-11-06

**Authors:** Luca Botturi, Tiffany Fioroni, Chiara Beretta, Davide Andreoletti, Alessandro Ferrari, Felipe Cardoso, Anna Picco-Schwendener, Suzanna Marazza, Silvia Giordano

**Affiliations:** 1 Scuola universitaria professionale della Svizzera italiana, Manno, Switzerland; 2 Università della Svizzera italiana, Lugano, Switzerland

**Keywords:** Privacy awareness, Personal data, Digital competences, Instructional design, Simulation

## Abstract

Privacy is a central issue in the digitalization of society and directly concerns all Internet users. Privacy education is part of the picture of a more just digital society: it aims at making users more aware of the importance of their data and of the technical and financial tools and processes that involve their personal data. Nonetheless, privacy education is confronted with a paradox: while people perceive the importance of privacy, they seldom take action to actually protect their personal data. iBuddy is a narrative simulation-based session inspired by research evidence about the privacy paradox and aims to (a) enhancing awareness and (b) promoting the uptake of privacy-safe behaviors for secondary and higher students (age range 11–20). The paper presents the design and development of the simulation and of the following modular debriefing, as a case study in evidence-based collaborative instructional design and in the instructional used of digital technology. The evaluation of iBuddy, which combined a post-session satisfaction and perceived learning survey (*N* = 978) and a follow-up survey (*N* = 124), provides insights in the novel domain of privacy education. Results suggests that iBuddy sessions are engaging, effective and conducive to medium-term behavioral change, thus indirectly confirming the design assumptions about how to tackle the privacy paradox through a simulation-based approach.

## Introduction

Online services and social networks mediate a large part of the time we spend online: searching for information, texting, sharing pictures, reading the news, watching videos, buying goods mostly happens on commercial platforms such as Google Search, Instagram, YouTube, Amazon, TikTok, or newspaper websites, etc. Most platforms recognize us thanks to a nominal account or via cookies, so that they can offer us improved and customized services; some of them also use our data to target us with personalized advertisement. In both cases, during any online interaction we leave digital traces that contribute to enrich one or more of our digital profiles (Eke et al., 2019).

Today personal data and digital profiles are a source of wealth. Digital traces can be used, with more or less accurate results, for example to monitor healthcare (Kogan et al., 2021), to steer marketing campaigns (Airoldi, 2021), to predict voting behaviors (Bach et al., 2021), so that data is more and more the new oil (Humby, 2006) fueling platform capitalism (Srnicek, 2017). Nonetheless, the processes by which our personal data are collected, stored, elaborated and used still remain largely invisible and unknown to most people.

Data privacy concerns “the claim of individuals, groups, or institutions to determine for themselves when, how, and to what extent information about them is communicated to others” (Westin, 1967, p.7), and is tightly connected with freedom and justice (Kimmel, 1988). Privacy is a central issue in today’s digitalization of society and has become a topic in digital competences frameworks (Carretero et al., 2017; UK Dept. of Education, 2019). Personal data management, identity management and data protection are, under different names, key constituents of active digital citizenship (Carretero et al., 2017; Guitert et al., 2017; JISC, 2014).

What is clear is that we cannot expect privacy issues to be magically fixed by any off-the-shelf technical or legal solution. Data monopolies offer services that are not easily replaceable, they have huge information channels that can be used to give a fudged version of the use of data they do and of the related privacy risks, and, last but not least, are economically very powerful. While technical, legal and political solutions are being discussed in the proper venues, we support a focus on education, relying on the idea that, in order to mitigate the impact of data privacy issues, everybody has to play an active role, including the final users, and in particular young people. Educating young people does not only mean to protect them, but also to raise a more aware future generation of innovators and decision-makers.

Between 2019 and 2021, we took part in an initiative aiming to rising awareness of threats of data leakage and related risks for smartphone users and all users that interact with IoT (Internet-of-Things) devices, with a focus on young people (age groups 12–15 and 16–20), educators and policy makers in Switzerland (Giordano et al., 2020).

In this paper we present and discuss the design, development, delivery and assessment of the iBuddy session, a 2-hour interactive class simulation-based session developed to let young people wonder about personal data capture and storage and to stimulate learning and privacy awareness raising.

The purpose of the paper is threefold. First, it presents iBuddy as a case in evidence-based collaborative instructional design process, focusing on teaching and learning *about* digital technologies. Second, it describes the iBuddy system, documenting a possible implementation of and simulation-based teaching and learning approach *with* digital technologies. Finally, evaluation data provide evidence to support insights in both privacy education and in simulation-based learning.

The next section outlines the state of the art in privacy awareness education, while the following one illustrate the instructional design of the iBuddy and of the related app and server system. The third section presents the assessment data collected in 52 school classes and identifies the main strengths and weaknesses of the system and of its integration into the proposed learning activity. The final section proposes some conclusions and outlooks for further work.

## Privacy awareness: state of the art

### Privacy awareness

While the majority of users declare to be *concerned* with privacy risks, research has demonstrated that most of them do not behave consequently as they are not really *aware* of them (Andreoletti et al., 2020; Ferrari et al., 2015; Zyfallari, 2021). Users grant data access to apps that gather a huge amount of personal and sensitive information, often considerably more than they actually need for the services they provide (Agarwal & Hall, 2013; Felt et al., 2011; Vidas et al., 2011). In many cases, people lack the ability and knowledge to manage their privacy (Aditya et al., 2014; Park & Jang, 2014). Furthermore, people are rarely aware of data regulations and of the value of their data (Acquisti et al., 2016; Steel et al., 2013), but when they get informed about the importance and value of their personal data, the risks related to data exposure and how to address them, many of them take action (Zyfallari, 2021).

A 2019 survey with 21,203 respondents between 16 and 64 years from 21 countries reported that in some countries like India, Germany and Great Britain awareness has grown above 50% of the population, but several other countries, including the USA, China and France, seem to lag behind (Statista, 2019). A survey from IBM-Harris (2020) conducted in the USA shows that consumers are demanding to understand and have control over where their data go. These numbers echo the findings of a survey by DuckDuckGo (2019) that found that about 80% of the contacted people have modified their privacy-related settings on their social media accounts or reduced their social media usage to feel more protected. Additionally, a significant amount of people (almost one fourth of the participants) abandoned a social media profile due to privacy concerns. This tendency is further confirmed by a report by Cisco (2019) that indicates that new generations are progressively becoming more attentive to their privacy.

Despite the evidence of a general increase in awareness of privacy issues, it has been widely observed that Internet users still tend to accept the disclosure of their private data in exchange for some rewards. Although data have an economic value for the platforms that gain access to them, the nature of such user rewards is mostly symbolic, such as popularity in online social media, thus generating a peculiar asymmetry.

### The privacy paradox

The inconsistency between the individual notion of privacy and the observed behavior is commonly referred to as the *privacy paradox* (Barnes, 2016; Norberg et al., 2007). In a literature review, Kokolakis (2017) shows that there is no agreement on the actual existence of the privacy paradox. Indeed, while some works provide evidence of it, others deny it. For example, some studies (Hughes-Roberts, 2013; Reynolds et al., 2011) illustrate that a set of students makes use of online social media in a far less cautious way than what previously declared. Conversely, other studies provide evidence that young people declaring privacy concerns actually enforce countermeasures, such as restricting access to their profiles and deleting pictures whose release might harm privacy (Young & Quan-Haase, 2013).

Kokolakis (2017) also tries to move beyond such contradictory results and provides at least three interesting interpretations, which we used to determine our instructional approach:

1. The first interpretation goes against the existence of the privacy paradox. According to this view, the proponents of the privacy paradox often use the wrong methodology to assess the level of awareness of privacy risks. In particular, it is argued that surveys are not suitable to effectively capture users’ behaviors. Based on this interpretation, most people only have vague privacy concerns and are not really aware of the actual privacy issues affecting social media. In our view, privacy concerns should be assessed by monitoring the actual behavior of users, instead of only asking their opinion. A possible approach is to collect the sensitive information that users disclose and to let them compare this data with their preconceived privacy concerns.
2. The second interpretation also denies the existence of the privacy paradox by arguing that users have incomplete information about the risks connected with their behaviors. Based on this view, despite people having severe privacy concerns, they do not actually know that their digital behavior leads to exposing privacy-sensitive data. The issue at stake here is pretty informational: users should understand how their data is being captured, stored and used, by whom and to what purpose—in a word, the invisible flows of data collection and processing and their related economic effects should be made somehow visible.
3. A third interpretation is that the privacy paradox does exist, and it can be explained considering that users have a positive privacy vs. utility trade-off. In other words, whilst users acknowledge the existence of privacy risks and have enough information, they consider that the benefits of disclosing sensitive data outvalue these risks (e.g., popularity among peers is more important than protecting private data). The issue here is about balance: what am I ready to pay in terms of personal data for the services I use? In our view, critically understanding the incredible financial power of the Big Tech and thinking in the long term (e.g., that pictures posted today may compromise a job interview five years from now) are part of the game here.

It is likely that different users or user groups have different experiences of the privacy paradox, and that all three interpretations (and maybe others) can actually explain the behavior of some users. In any case, it is important to acknowledge that the conceptualization of *privacy risks* is inherently challenging, as this depends on a mix of technical, cultural and subjective factors (Kokolakis, 2017). It is nearly impossible to extensively identify all the possible dangerous digital behaviors and to objectively measure the associated risks. While the theoretical debate is yet open, it is clear that enhanced user awareness is central in the discussion (Deuker, 2011).

We translated these insights into a practical instructional approach implemented in the iBuddy sessions: providing participants with an opportunity to experience first-hand the effect of the exposure and capture of personal information that they were not aware of having exposed. iBuddy aims to generating a surprise effect or, as we labelled it, a *wow effect* (Kamstrupp, 2016).

### Sensitiveness of privacy risks

Pursuing the *wow effect* required identifying specific situations that would be perceived as risky by most users. A measure of privacy risks can be obtained by combining two main factors: sensitivity and visibility of data (Aghasian et al., 2017).

1. *Sensitivity* measures the level of confidentiality of some data. Several studies (Madden et al., 2014; Schomakers et al., 2019) provided a categorization of data into different levels of sensitivity. For example, the bank account number or the digital signature are considered extremely private. Locations, pictures, and online dating activities are seen as moderately private. The e-mail address and political affiliation are instead regarded as the least private data.
2. *Visibility* measures the ease of access to the data, and it is further articulated into (a) accessibility, (b) difficulty of extraction, and (c) frequency of occurrence. For example, data can be accessible to the owner only, or to friends, or even be publicly available. Data can be very structured (e.g., metadata on users’ profiles) or unstructured (e.g., a picture). Then, data can be frequently exposed (e.g., age is repeated on every social profile of a person), or rarely exposed (e.g., a specific interest is only mentioned in a single post).

In our work, we started from the aforementioned conceptualization as a guideline to generate the *wow effect* (Kamstrupp, 2016). In particular, we tried provoking students with data that are highly sensitive, difficult to extract (e.g., from unstructured and not easily accessible data, such as pictures in their smartphones’ gallery), and which are not frequently exposed.

### iBuddy Session Design

The Making A Privacy-Aware World (MAPAW) project was developed to promote personal data protection awareness in young people (from lower secondary to higher education, age range 12–20) based on two assumptions that wrap-up the discussion reported in the previous pages:

1. Deep awareness can only be based on factual knowledge and information (Botturi, 2004), i.e., young people do not only need list of “do’s and don’t’s” but need to understand how technologies operate in order to make informed decisions.
2. The actual relevance of data protection will only be perceived in relation to personal situations in which sensitivity and visibility are at stake, and not as generic broad social issues.

Based on such assumptions, we set forth to develop a set of teaching and learning materials for different school sectors and class scenarios. The iBuddy session, an interactive 2-hour class simulation which implements the wow-effect approach presented above, represents the project’s main instructional achievement.

### Instructional design process

The instructional design process was set up as 2-tier collaborative design, loosely inspired to the R2D2 model (Willis & Wright, 2000). The *core team* brought together experts from different fields, i.e., educational technologies, digital networks and digital law. The team, coordinated by the first author of this paper, developed design proposals, which were then discussed and refined with the *peer-council*, a group of 15 teachers and students representing the main target groups, who were asked to provide feedback at different stages. The ARCS model (Keller, 1987) was taken as reference for the motivational design of the session.

At the outset of the design, the team agreed on respecting some constraints, which are usual in the Swiss school settings, namely:

1. Students would attend the session as a class (not in smaller groups), which means a group of 15 to 25 pupils.
2. A regular session should last exactly two class periods, which means from 90 to 100 min depending on the school daily schedule.
3. From a technical point of view, the implementation of the session should only count on the availability of an overhead projector, and nothing else.

**Fig. 1:**
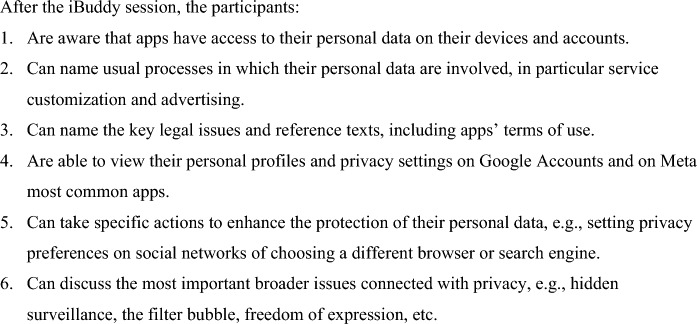
iBuddy session learning goals

The first step in the instructional design process was the definition of learning goals, which of course had to be adjusted to the different grades. The core team defined the learning goals presented in Fig. 1, which were then fine-tuned and approved by the peer council. Learning goals 1–3 focus on awareness and were set for participants of all ages; goals 4–5 focus on action, and were set for participants of age14 and above, which most commonly have a personal smartphone with unrestricted account; goal 6 is about societal and cultural issues and was considered in sessions with participants of age 16 and above.

### Simulation as a safe environment

Already at the outset of the design phase, a dilemma was identified: on the one hand, we were aware that, in order to make privacy issues relevant, we had to address students’ personal experiences and work with their data, so to somehow push them out of their comfort zone; on the other, we were aware that it would have been difficult to discuss real and possibly delicate personal situations in class. We needed to draw a magic circle in which students would be able to perceive the effects of personal data breach in their own experience without generating embarrassment or panic.

The solution was to transpose the activity in the fictional world of a narrative-based simulation, in which students would enter a risk-free game-like environment (Botturi & Loh, 2009; Rudolph et al., 2014). Simulation games are a form of experiential learning in which the learner is immersed in a simulator (Hale Feinstein et al., 2002)—in our case, a network system that extracts personal data from personal digital devices. Simulations seem to support the transfer of learning to real life (Deshpande & Huang, 2011), and this corresponded to our second set of learning goals.

The participants of an iBuddy session are invited to install the iBuddy app and take part in a sci-fi-like endeavor: configuring an AI-drive carbon-body android that will join their class in a few weeks. The process and the app have a ludic character, and the simulation is carried out in a playful environment. During the simulation the participants’ personal data become part of the system and are used to talk of somebody else—namely, the new android classmate, thus fostering vicarious experience and avoiding direct focus on the participants’ experience.

The learning impact of a game-based session (including simulations-based) strongly relies on the feedback (Hays, 2010) or debriefing (Babazadeh et al., 2022; Betrus & Botturi, 2010; Peters & Vissers, 2004) that the instructor provides. So, we set out to design also a proper debriefing session to integrate the simulation experience and integrated “cliffhangers” or pointers to the debriefing already during the design of the simulation (Peters & Vissers, 2004).

### Design, implementation, fine-tuning

Based on the identified learning goals and on the idea of creating a safe space, the team decided to design the session in two phases: (a) a highly engaging narrative-based playful simulation based on interactive technologies that could be played with participants of all age groups; and (b) a flexible modular debriefing, which could be adjusted to the age group and to the questions and ideas that would emerge in each individual session.

The core team developed a first outline of the simulation and illustrated it visually on a storyboard, in order to facilitate the discussion with the peer group, which provided feedback both on the simulation itself and on the topics that could be addressed in the debriefing phase.

The core team developed then the required software and instructional materials and tested a beta version of the iBuddy session with 12 classes. The collected feedback from pupils, teachers and instructors was revised with the core team and the peer council, which led to revisions of both the session structure and specific instructional materials.

The following section presents the final outline of an iBuddy session, which is articulated in the following 5 phases, including both the game-based simulation and the modular debriefing.

## iBuddy session outline

### Phase 0. Introduction (duration: 20′)

An iBuddy session is led by a presenter, who greets the students and introduces the narrative, which is central to engage students to willingly play (Botturi & Loh, 2009) and to establish a fictional contract (Diekmann et al., 2012; Muckler, 2017). The presenter also asks the students to keep the content of the session confidential: on the one hand, this captures their attention, on the other, this will be reprised at the end of the session as a formal request not to spoil the session to the other classes anticipating its actual content. The presented uses the following textual guideline:

“Welcome! Before we start, I need you to agree to keep secret what you will experience in the next two hours. In fact, your class has been selected among many for a cutting-edge technological experiment. In a few weeks, you will welcome a new classmate—but he (or she!) will be special. Indeed, s/he will be a synthetic buddy, an android with advanced carbon biotechnical body animated with sophisticated artificial intelligence algorithms. This new buddy will blend in and make your class and your school much better. Our task today is to configure your new artificial buddy: determine if it is a boy or a girl, his or her tastes, and so on. In order to do this, we will use a special app”.

Students are then invited to download and install the iBuddy app, which is a regular Android and iOS app. If some students do not have a smartphone or do not have the permission to install new apps, they are invited to work in pair with a classmate.

After the installation, the app remains in stand-by, waiting for the presenter to unlock the session.

### Phase 1. Creating a profile (duration: 10′)

Once everyone has the app installed (or sits beside somebody who does), the presenter unlocks the session. The app asks for permission to access the picture gallery and the contact list. Of course, students can do as they like; in case they ask, the presenter will only say “Feel free to do as you usually do in such cases”.

**Fig. 2:**
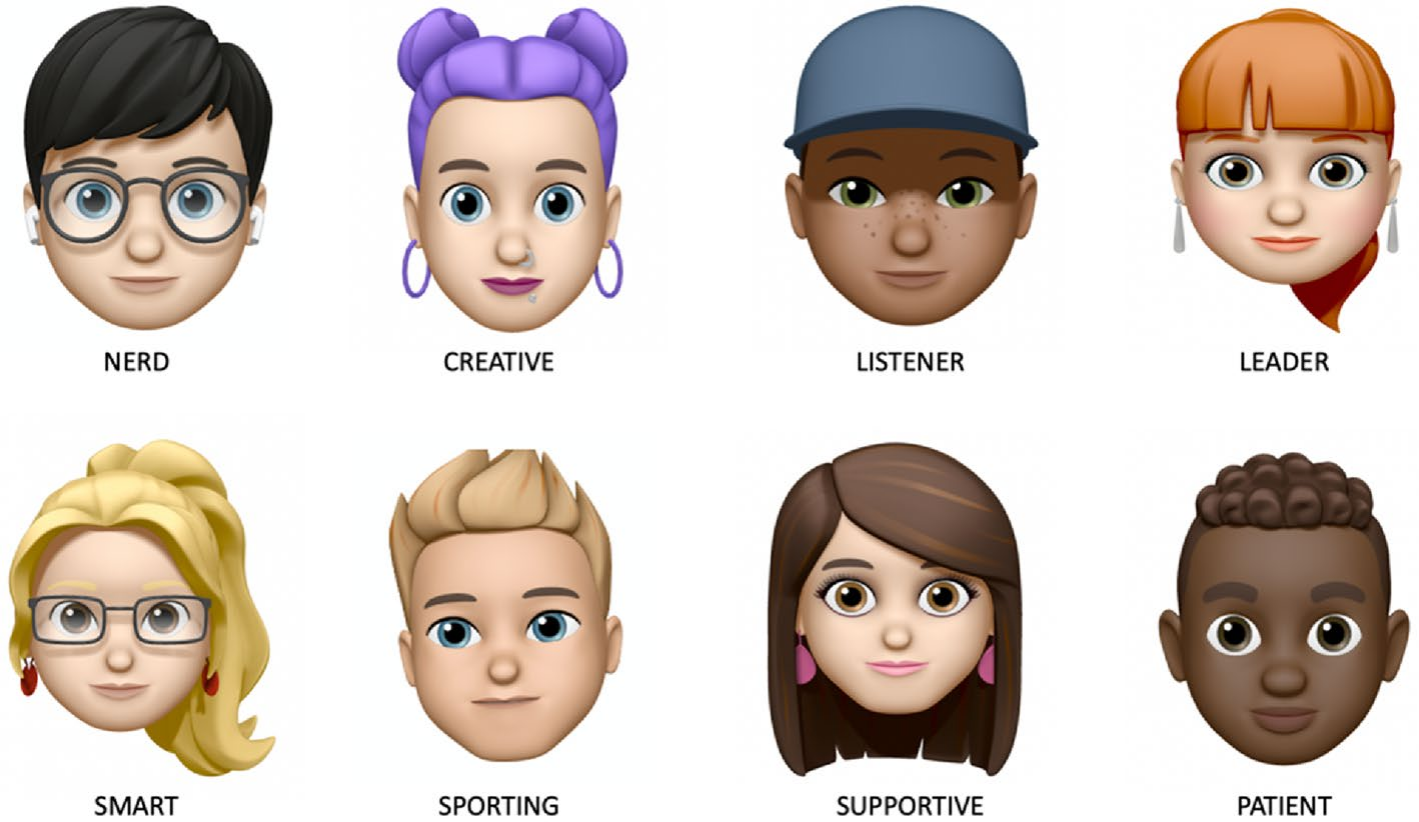
Screenshot of the basic buddy profiles roster

The app then asks a few personal information items, like a nickname (the presenter clearly indicates *not* to enter real names), favorite food and sport, and to add a profile picture. After that, eight *basic buddies* are presented, i.e., standard androids with basic features like gender, race and one personal characteristic, e.g., “supportive”, “sporting”, “smart”, etc. To emphasize the game-like nature of the simulation and to avoid potential direct resemblance to actual classmates, the basic buddies are represented in cartoon style (Fig. 2).

While students create their app profile, the iBuddy system performs a personal data collection process in the background, copying to the local server a sample of 6 pictures from the smartphone gallery, the contacts list, data on installed apps and the associated usage time. Of course, some data will only be accessible if the participants granted access to them. An *operator*, usually sitting at the back of the room and often unnoticed, is able to view the collected data.

### Phase 2. Class questions (duration: 10′)

This phase is where the wow-effect is generated. Once everyone has picked their favorite basic buddy, the app goes to stand-by and the presenter informs them that the system will now ask some questions that will require a collective answer from the whole class. The questions are displayed with an overhead projector and, although the look they same to the students, the system displays two types of questions:

1. *Standard* questions are ready-made and generic, and include items like “What is your favorite subject?”, or “How many of you have a pet at home?”
2. *Custom* questions are created on-the-fly from templates by the operator using the personal data collected from the participants. Custom questions might include items like the ones presented in Table 1.

**Table 1:** Sample custom questions

Data used	Sample custom question
Installed apps	15 of you use Whatsapp. Shall your new buddy also use this app?
Usage data	On average, you use TikTok 30 h every month. This is about 1 h a day! Will your new buddy also be such an eager tiktocker?
Contact list	Manu has over 215 contacts in her phone. Will your new buddy also be so popular?
Contact list	Johnny is called “Magic J” in somebody’s contact list. Will your new buddy also use such fancy nicknames?
Picture gallery	Deb seems to like pets like this one. Will your new buddy also share this passion?

The presenter is able to mix standard and custom questions on the fly according to the reaction of the class. Usually, 3 to 5 custom questions are enough to generate a deep and rich discussion in the following phases. Seeing supposedly private data displayed to the whole class generates a powerful wow-effect, even if in a safe environment. The session is always concluded with one or two standard chill-out questions.

The operator is of course prepared to select effective but safe information from the available personal data, for example excluding embarrassing (or illegal^1^) content. This is particularly important for pictures, which are perceived by teenagers as their most intimate data. Based on experience, effective pictures (i.e., that easily generate a wow-effect and that are safe) include portraits of the students in normal situations (e.g., smiling, or walking), pictures in which people appear but are not identifiable (e.g., on a landscape, or from the back), and pictures of pets and food. Other pictures have potentially embarrassing subjects, and would definitely generate a wow-effect, but should be considered unsafe to be used in class. They include portraits of people other than the students and portraits of the students in unusual situations (e.g., doing faces, dancing, with strong make-up, etc.). Finally, pictures of landscapes or objects are usually ineffective, i.e., they do not generate any wow-effect).

**Fig. 3:**
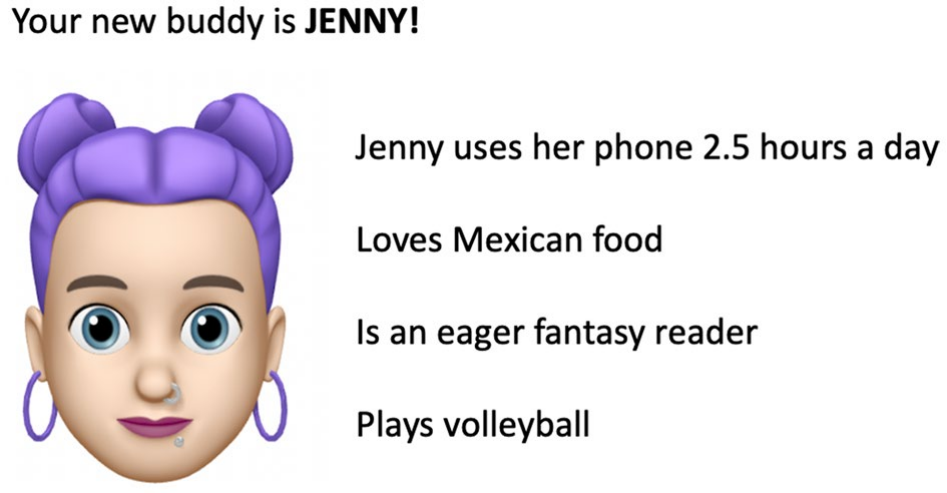
The presentation of the customized buddy

Eventually, the system then generates the perfect iBuddy profile, based on the answers provided in phase 1, and displays it to the class (Fig. 3). While the artificial buddy profile is the MacGuffin that sets in motion the whole session, the actual result, which is often awaited for with great thrill, is not that important for learning.

### Phase 3. Debriefing part 1: behind the scenes (duration: 10′)

After the class saluted their future artificial buddy with a round of applause, the simulation is over. Like all simulation-based and game-based sessions, what happens afterwards determines the actual learning (Betrus & Botturi, 2010; Peters & Vissers, 2004). The debriefing of an iBuddy session is articulated in two steps and each of them is articulated into short modules that allow adaptation to the specific age group and class.

The first step in the debriefing always includes three modules (1.1, 1.3, 1.4) and an optional one is available (1.2).

*M1.1—What happened* The students are asked about the simulation, so that they can identify and share the “tricks” that let the system generate the custom questions. In most cases, and especially with younger students, they noticed that the system captured pictures from their phones but seem to find it normal that their contact and app lists were being read.

*M1.2—Dealing with emotions* Individuals or the whole class may feel strong emotions about what happens. Some might even feel threatened or scared that their private information could be revealed. In such cases the presenter takes time to discuss such feelings and re-establish a positive environment, also relying on the information that s/he will present in the next module.

*M1.3—Technical features* The presenter explains how the app transmitted their data to the local server and says that this is what many common apps and services do. A special screen from the system allows the presenter to show the collected data (e.g., the number of pictures or contacts). With older students, reflections can be made on how such data can be used by AI algorithms to extract or generate additional details.

*M1.4—Data deletion* Eventually, the presenter clicks on a special “red button” on the system interface, that deletes all collected personal data from the server and provides visual cues of the operation.

### Phase 4. Debriefing part 2: the internet today (30′)

The second step in the debriefing aims to explore how personal data collection, storage and elaboration happens in the real life. The focus is on regular and legal uses of personal data (e.g., personalized advertisement) and not on malicious uses (e.g., grooming or identity fraud), and the path followed largely depends on the questions asked by the participants. For this part, the presenter is equipped with flexible learning modules (each lasting 10 to 15 min) and the related learning materials, that enable her/him to introduce activities on the fly, following the questions of the class and targeting the specific objectives for the age group.

*M2.0—How the internet works* In some classes students only have a rough idea that the Internet is a network (and not an app) and have no idea that their data can be copied and transferred to servers in different parts of the world. A simulation is available to let the younger student grasp how basic internet technologies work.

*M2.1—Service personalization* This module provides reflection on how digital apps and services can provide customized information (e.g., posts selection) to their users. A group activity simulating Google Search is available for younger students. For older students the module can be expanded with a discussion of filter bubbles and their personal and social effects.

*M2.2—Advertising* A common business model for many big tech companies is based on targeted advertising. This module uses example for understanding how it works (and how it is different for example from sponsoring or philanthropy) and how it can be adapted to the digital world.

*M2.3—How much are your data worth?* This module is about the economic value of personal data, which is often hidden in our daily use of “free” apps. With older students, key figures from the balance sheets of big tech companies are compared to the GDP of European states and used to spark the discussion. For higher education students, the module can be expanded to integrate a discussion about the power of digital monopolies.

*M2.4—Know your profile* This module provides indications about how we can check our digital profiles in most common apps and services (including Meta and Google) and on how we can adjust our privacy settings.

*M2.5—Terms of use* Reading and understanding the Terms of use as a legal text is a basic good practice for privacy awareness. A group activity based on a detective story is available to engage the younger students. With older students the discussion can also include national and international privacy regulations.

*M2.6—Alternatives* This module is about privacy-safe tools for everyday use as alternative to mainstream tools, including DuckDuckGo, Firefox, Signal, etc.

Depending on the age group, the questions asked, and the time available, the presenter is free to adjust on-the-fly a specific path for this phase of the debriefing, combining usually 3 or 4 modules.

**Fig. 4:**
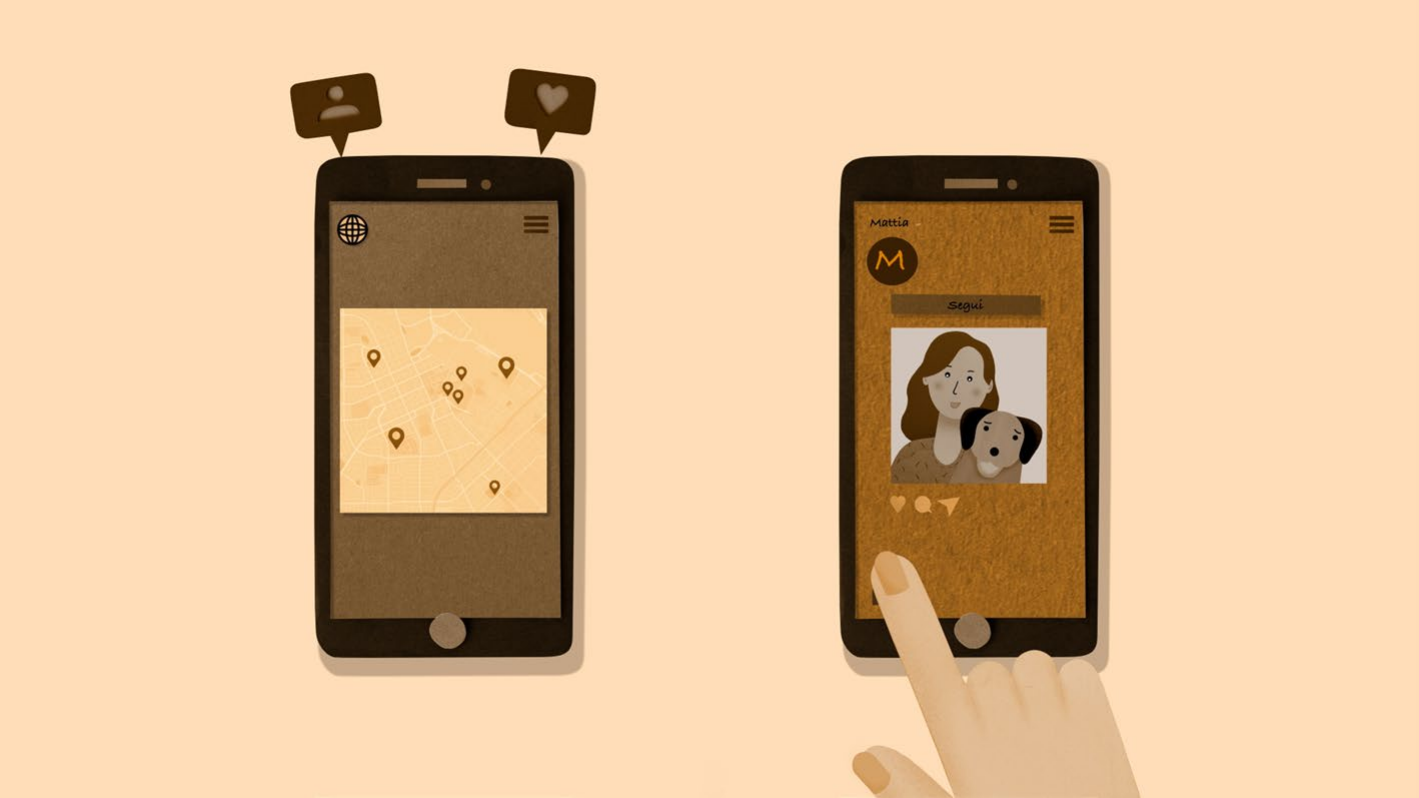
A screenshot from Emma’s video set

Within the MAPAW project a set of 4 short digital stop-motion video clips (duration 2′–3′) were developed and released under Creative Commons license. They present Emma, a girl, and her use of a social network called Momentum. The videos address personal data collection, personalized advertising, the technical network infrastructure involved in data collection, and legal aspects (Fig. 4) and can be used to wrap up the discussion.

All original materials are available at www.protectyourdata.ch in Italian and other Swiss national languages, along with pointers to other useful online resources for privacy education.

## Technical, legal and ethical issues

### Technical architecture

We briefly outline here that the technical architecture of the iBuddy system, which was devised in order to make iBuddy a smooth, entertaining and technically safe session. It can be described from the functional and physical points of views.

**Fig. 5:**
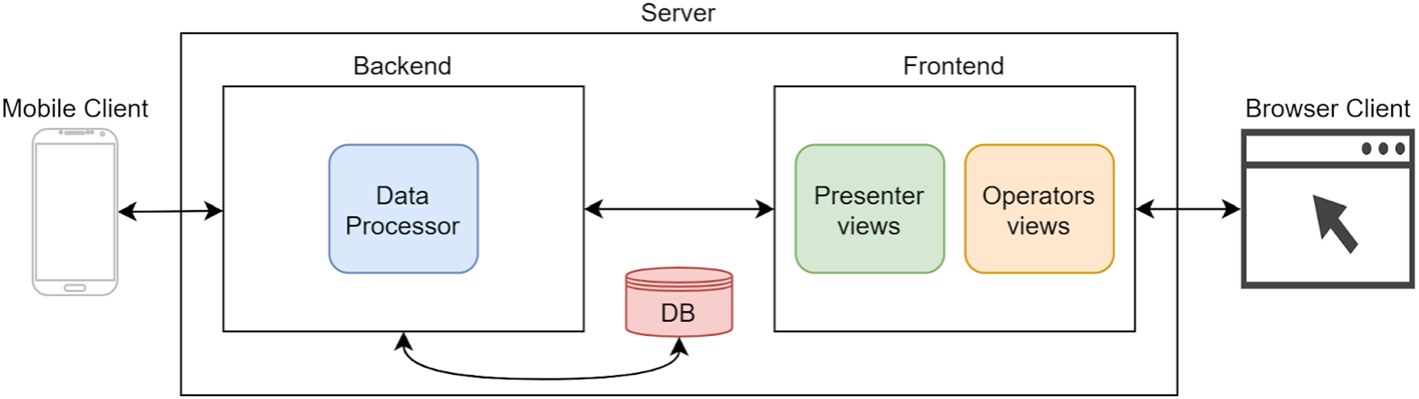
iBuddy functional architecture

The functional architecture, depicted in Fig. 5, is composed by three parts:

- Mobile Client: mobile application that collects data during the experience.
- Server: local server that receives and processes all the collected data.
- Browser Client: web accessible resource that exposes several views.

**Fig. 6:**
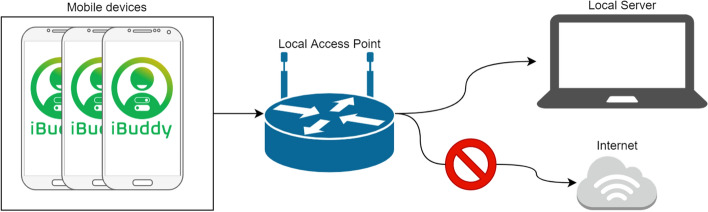
iBuddy physical architecture

From the physical standpoint, as shown in Fig. 6, the architecture includes at least the following devices:

- Mobile devices: user’s mobile phone(s), where the user runs the iBuddy client.
- Local Access Point: a device with NO access to the internet that manages the local network.
- Local Server: a desktop or mobile computer where the iBuddy server runs.

Extra devices may be needed for the presenter and the operator. Usually, the operator works directly from the Local Server while the presenter uses a different machine, with split screen, one private (where s/he can view and activate the questions) and one connected to a class beamer.

The users’ mobile phones and the server are connected to the local network provided by the local access point, and the server and the network are isolated by the rest of the world (Internet) to avoid any unauthorized access-to or leak-of the data collected during the experiment.

### Ethical and legal aspects

During the design of the iBuddy session, we faced a number of challenges, not least trying to generate the wow-effect while minimizing interference with the user’s private sphere. Nonetheless, collecting students’ data from their smartphones, even if for teaching purposes, can seem ethically extreme. Indeed, this is exactly what happens daily with most commercial applications and web services—we only made the process visible by completing it in a short time and within the classroom. The iBuddy system was carefully designed to meet the legal requirements of the Swiss Federal Personal Data Protection Law and to respect ethical standards.

First, we needed to understand to what extent it is legally possible to collect personal data for teaching purposes, given that iBuddy targets pupils aged 12 and over. In Switzerland there is no clear legislation foreseeing a minimum age for consenting to data processing, which depends on “discernment capacity”, which might be subjective. As a solution to this issue, we developed a clear and full explanation of the iBuddy app and the way data are processed throughout the simulation for the school’s headmaster and for the teacher that is in charge of the class and who must be on site during the simulation. After having received this written explanation, the headmaster is free to accept to go forward with our activity in the school or in a specific class.

Moreover, each student is completely free to access the privacy policy properly attached to the iBuddy app and to grant separate and specific consent for access to the photo folder and the contacts list of her/his phone. Without explicit consent, the operator is not allowed to process any photo and/or contacts list, the system not being even able to collect them. During the class interactions made so far, part of the pupils granted their consent while others denied it (with varying proportions in different classes), and the presenter took this as an opportunity to discuss the consequences of granting and denying consent for data processing.

When starting the simulation, the app prompts the user to write a nickname which will be connected to the personal data collected from that phone. The choice of a nickname instead of the actual name (which is reiterated by the presenter) was done so that neither the presenter nor the operator can connect the data to any subject.

A clear privacy policy can be accessed anytime from the app. Even if the aim of the simulation is to achieve a wow-effect, we published a proper easy to understand privacy policy to comply with the law, and part of the debriefing is indeed also about going through it in order to raise awareness about this important but often neglected text, which lets users understand if, how and what personal data are processed when exploiting an app.

As mentioned above, in order to keep personal data secure once they have been collected, these are kept in a single copy on a local server with no internet connected, so that no interference with unauthorized third parties is possible. Last but not least, all personal data are completely deleted during the session itself, which usually means about 30 min after their collection.

## iBuddy session assessment

### Sessions and informal feedback

Between March 2021 and March 2022, 52 iBuddy sessions were carried out, engaging a total of 978 students in 17 schools, as illustrated in Table 2. In many instances, the activity was embedded in thematic educational units about technology and data privacy. The first 12 sessions, scheduled in Spring 2021, were used to test the system and to fine-tune both the app and the class materials thank to the feedback provided by the peer council.

**Table 2:** Number of schools, classes and students that took part in the iBuddy project by school level

	Secondary school		High school	University	Total
Age range	11-12y	13-14y	15-19y	20+	
Classes/groups	20	11	17	4	52
Students	344	219	343	72	978
Schools	10		6	1	17

Both the students’ and the teachers’ informal feedback during and after the sessions was positive, at times enthusiastic. All classes engaged deeply with the simulation, and at the end of the session many students in the lower age groups asked if they would really welcome an android buddy in their class, or if he or she would “also eat and talk like us”. In most classes the students asked many questions during the debriefing, also bringing examples from their personal experience.

### Assessment method

The evaluation of the iBuddy experience was aimed to answer the following questions: Did the participants enjoy the iBuddy session and found it engaging? Did the participants learn something useful about privacy and data protection? What is the impact of an iBuddy session in the medium term?

While a pre-post survey design, possibly including an objective competence assessment, would have provided more reliable and comprehensive data, its implementation would have generated a priming effect that would have likely spoiled the simulation. We therefore decided to collect structured feedback in two distinct moments: (a) at the end of the session, in order to assess the participants’ satisfaction and perceived learning; and (b) after two months, in order to explore the medium-term learning effect of the iBuddy session.

The end-of-session survey was administered during the last 10 min of every session and contained 4 closed and 4 open items. In the following analysis we consider the data from the closed questions.

The evaluation items were the following:

1. How much did you enjoy this activity? (Enjoyment)
2. How useful is this activity in raising awareness among young people about a responsible use of their personal data? (Usefulness)
3. How involved did you feel in these issues? (Involvement)
4. How important do you think the topics are? (Importance)

The participants were invited to express their feedback rating each item on a 1–10 Likert scale, where 1 corresponded to the lowest score and 10 to the highest (no intermediate points were labelled). Students could also leave a textual comment for each question. The data were analyzed using descriptive statistics.

While an engaging class simulation goes rarely without appreciation, we wondered about the medium-term effect of iBuddy: do students remember what was presented in the relatively short session and change their behavior? To shed some light on these questions a short follow-up survey was distributed 2 months after the session. At the time of writing this paper 563 participants were contacted, and 21% responded (*N* = 124; 57 males and 67 females). The follow-up survey includes 7 sentences starting with “After the iBuddy session…”:

1. I pay attention when I install a new app.
2. I read or at least screen the terms of use of the apps I install.
3. I check and adjust privacy setting for my social network accounts.
4. I select what I post or share on social networks.
5. I think about why some ads appear when I browse the web.
6. I check and maybe delete cookies on my devices.
7. I am generally aware about my personal data.

The respondents could express their agreement or disagreement on a 3-point Likert scale in which is 1 corresponds to “like before”, 2 to “somewhat more than before” and 3 to “much more than before”. The data were analyzed with descriptive statistics.

### End-of-session results

All participants across all school levels found the topic of data privacy *very important* (*M* = 8.96; *SD* = 1.36), the activity *very useful* (*M* = 8.58; *SD* = 1.52) and *enjoyable* (*M* = 7.82; *SD* = 1.60), thus consistently suggesting that iBuddy sessions are effective (Table 3).

**Table 3:** Average scores for the four evaluation items (*N* = 978)

School	Enjoyment	Usefulness	Involvement	Importance
Secondary school (11-12y)	7.33	8.27	6.15	8.82
Secondary school (13-14y)	8.16	8.73	6.61	8.97
High school	8.04	8.54	8.05	8.98
University	8.13	9.21	8.65	9.53
Global	7.91	8.67	7.09	8.96

**Fig. 7:**
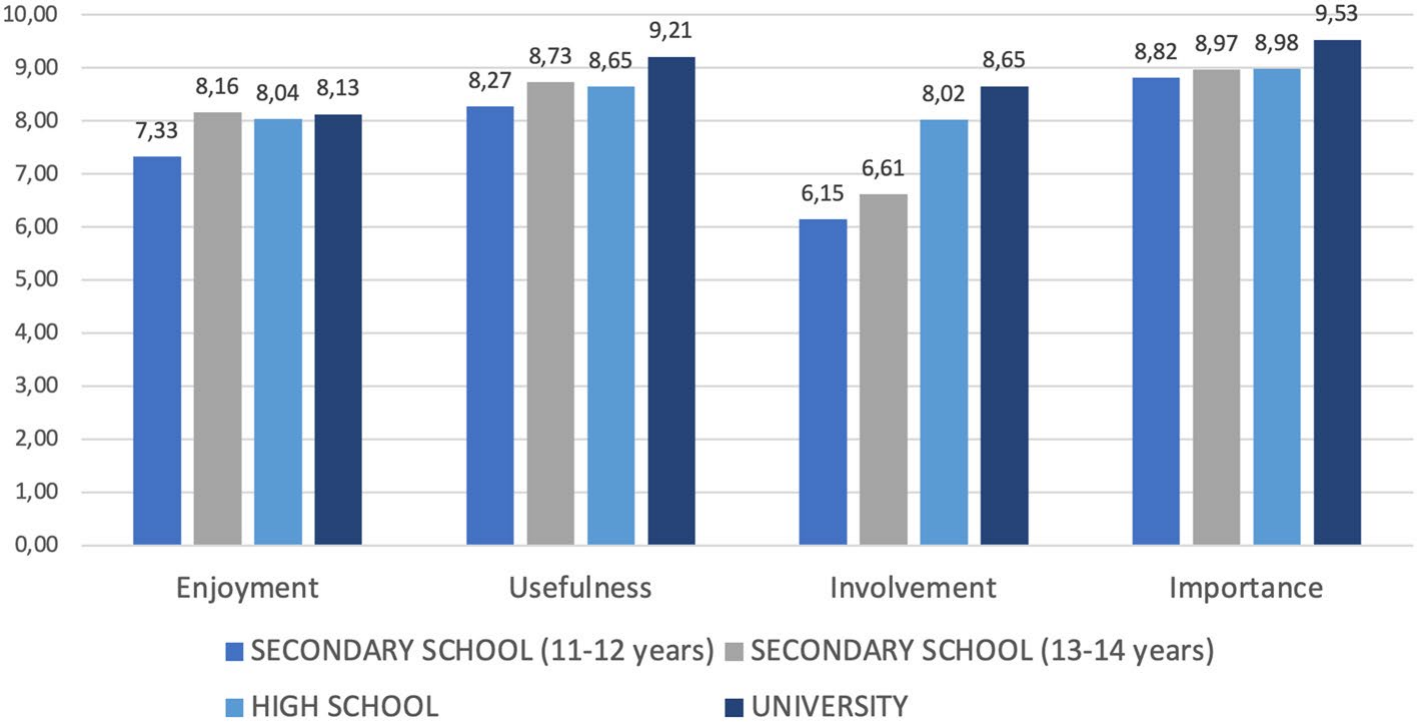
Average scores obtained in the various categories (*N* = 978)

School level differences appear on the dimension of involvement: younger participants perceive the crucial importance of the topic in general level but do not feel it to be personally relevant (Fig. 7). The global average score for *involvement* is lower, while standard deviation is higher (*M* = 7.09; *SD* = 2,18), and personal involvement increases with the age of the participants, from 6.15 in the secondary school (11–12y) to 8.65 in higher education. This can be explained by the fact that fewer secondary school students own personal devices, and they have less sophisticated usage practices than older students. In addition, many parents set restrictions on the use of their phone. Finally, young digital natives are usually less aware of the problems they can encounter in the web and perceive the use of their personal data by third parties as “normal”.

Open questions addressed the positive aspects of the session, its negative aspects, and the main message that participants brought home. Answers were mainly short (one sentence in most cases); they were read by two researchers individually, who then discussed together to identify the main emerging topics. The labels assigned to such topics were then used to tag each individual answers.

The analysis suggests that the most appreciated part in iBuddy sessions by participants from all school grades was the simulation with the creation of the artificial buddy (*N* = 444, 45.40%). Engagement (*N* = 173, 18%) and the development of awareness (*N* = 179, 16.97%) were the two other most frequently mentioned positive aspects. Only 336 participants (34.36% of the whole sample) mentioned a negative aspect of the session. Some participants (*N* = 113, 11.55%) felt personally touched when their personal data (especially photographs) appeared in front of everyone on the screens. Few participants (*N* = 72, 7.36%; especially younger students in middle school and high school students) mentioned that the session was too slow or too theoretical, and some (*N* = 57, 5.83%; especially younger students in middle school) did not appreciate the debriefing part.

The main message that the participants brough home is to raise the general level of attention when using digital devices and the Internet (*N* = 347, 35.48%) and awareness of how the Internet, social networks and BigTech companies handle our data (*N* = 209, 21.37%). Interestingly, and consistently with the follow-up data (see below), only one fourth of the participants mention specific actions that they can take to enhance the protection of their personal data (*N* = 234, 23.93%).

### Follow-up results

**Fig. 8:**
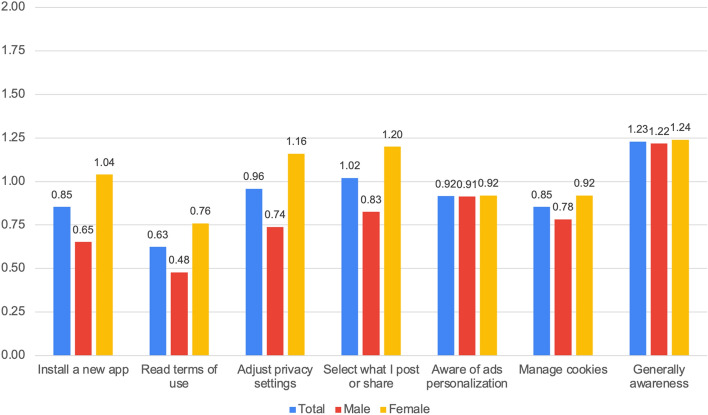
Follow-up survey data (*N* = 124)

The results of the follow-up survey are illustrated in Fig. 8. The highest score (1.21) is for the general item “I am more aware about personal data”; a slightly lower impact can be measured for the more specific items, which in any case received a positive level of agreement. This is of course expected and corresponds to previous findings. In general, iBuddy participants pay more attention to frequent active behaviors (managing privacy settings, selecting content to post on social networks, controlling cookies), and less for less frequent (selecting what apps to install and reading terms of use).

Interestingly, girls declare higher impact than boys on all items and across the whole sample. To further inspect this, we summed up the seven scores and calculated a unique indicator ranging from 0 to 14. Out of the 20 top-ranking participants (i.e., unique indicator score above 9) 14 are girls.

## Discussion and conclusions

In this paper we presented iBuddy, a simulation-based session for privacy education. We focused on its instructional design, based on research evidence, its technical design, and the assessment of its implementation with classes in different school sectors.

After 18 months invested in designing, developing, testing and fine-tuning iBuddy, results indicate that students of all age groups could positively engage with the simulation and completed the session with the clear perception of having learned. Follow-up data for secondary school students suggest that the impact of iBuddy sessions is relevant in the medium term and support the claim that iBuddy sessions are conducive of improved personal data safety behaviors, even if the uptake of specific data protection actions is still limited.

### Lessons learned in privacy education

Privacy education is a novel and yet non-formalized area of the curricula. In the design of iBuddy we could not rely on pre-defined content, learning goals or knowledge items. Privacy research studies outlined an educational need in relation to the so-called privacy paradox, and we tried to transform the perception of relevance of the topic into a decision for action. The iBuddy experience confirms that the privacy paradox can be tackled and possibly overcome with a focused instructional strategy (as opposed to generic frontal instruction), which in our case was implemented with a simulation.

The evaluation presented in this paper confirms that knowledge plays a key role in privacy education: about a half of the students indicated as the main learning from the session an increased awareness of how the internet, social networks and apps work, or indicated specific privacy-related behaviors. The follow-up data also indicate that the acquired knowledge provided good basis for developing a more reflective approach and safer practices. After learning how data is handled online, rules also make more sense. We believe this is likely to hold also for other areas of digital education in which ethical issues and personal awareness are at stake.

Our data indicate that only one fourth of the participants declared a change in behavior. This suggests that a single privacy awareness session is not enough to foster and support behavioral change.

Finally, the collaborative design process set up for iBuddy proved extremely valuable, bringing together experts with different profiles and a representative of all learning groups. Of course, the overall efficiency of the process was reduced, as gathering all the members and collecting their feedback took time, but it provided important confirmations or indications for change in a domain in which no pre-set path was available. We believe that the direct involvement of representative stakeholder—and not only of experts—is paramount in the design and development of instruction in new areas like privacy education.

### Lessons learned in simulation-based learning

The term *simulation* in iBuddy indicates a scaled-down safe and visible miniature-version of the data extraction and manipulation process that usually happens on a larger scale and invisibly online. In the terms of Hale Feinstein et al. (2002), the *simulator* is the iBuddy system, and the users play a role by entering the proposed storytelling circle. Instead of learning about it, students take active part in the process, and are offered for the first time a safe opportunity to see the data breach, capture and reuse in real time and in the same room, generating a *wow-effect* and a teachable moment.

While we all did our best to develop a suitable narrative for the iBuddy simulation and received positive feedback from the peer council, we were thrilled to see the reactions of the first classes that tried it out, as we did not know what to expect. To our surprise, no single student mentioned in the feedback survey that s/he did not appreciate or had problems engaging with the simulation; on the contrary, many mentioned it as the best part of the session.

To the instructional designer’s eyes, this sounds like one more confirmation of the power of a truly narrative setting. Students of course suspected that “the android classmate” project was a fake yet surrendered to the narration and suspended disbelief. This generated the game circle (Tekinbas & Zimmermann, 2003), or narrative circle, which made the experience light-hearted and playful: the participants suspended their worries and entered the game, accepting to be provoked until when the *wow-effect* was generated (Kamstrupp, 2016).

When their own data appeared in the simulation, students started to critically interrogate the technologies they use every day and to ask questions that go “behind the curtains” of personalized services and social networks. The wow-effect, although puzzling in the first place (as a few students remarked in the feedback survey), stimulated critical questions and created a powerful learning opportunity. The game circle made it possible to safely engage the participants until that moment.

Finally, the decision to use half of the session time for an in-depth debriefing, which was supported by the peer council, proved to be right. While this part of the session was of course less interactive and engaging, only a minority saw this as a problem and, in general, the students appreciated being able to discuss and understand what was previously only seen and felt. This confirms the importance of debriefing in game-based instruction (Babazadeh et al., 2022; Peters & Vissers, 2004). Moreover, the modularity of the debriefing phase, illustrated above, made it possible to adjust the activity to the specific class and situation, avoiding a one-size-fits-all approach.

### Limitations

While the outcomes presented seem to confirm the effectiveness of the iBuddy approach, they do not offer a sound basis for generalization.

First, we have no data about prior learning activities about privacy and online safety, either at school or in non-formal or informal settings; second, we do not have student profile data except age and gender, so that we do not know if impact varies across specific sub-groups; third, our sample was convenience-based (i.e., included students whose teachers organized an iBuddy session) and was limited to Switzerland, a European country with 99% penetration of mobile devices and high educational standards. How the same session might be adapted to different linguistic and cultural contexts is still an open question, as many contextual variables could impact its reception and impact. We also assume that local school cultures (including focus on digital competences) might be relevant.

All data presented is self-declaratory. While this does not raise any issues for the post-session assessment, it does for follow-up survey, where a more objective behavioral or competence analysis would be more appropriate. While this fell outside the scope of the MAPAW project, the development of an objective data safety scale would be a major advancement in this domain.

Finally, while the assessment data presented are rather extensive for post-session data, they are still limited for follow-up data. This will be improved in the upcoming months, when new subjects will be contacted for the follow-up survey.

### Outlooks

Our current work focuses on making iBuddy easily portable and replicable. The iBuddy system (app and server) supports multiple languages by design, and we plan to distribute the iBuddy system as open source soon. The development of a kit with a Raspberry-based plug-and-play hardware is also under discussion. Such technical instruments should of course be accompanied by localizations of the class materials (videos, activities, worksheets) and by the provision of proper training to presenters and operators.

From a research point of view a more thorough follow-up study about actual behavioral changes in the participants would definitely shed more light on the impact of iBuddy or of similar sessions sharing its instructional approach. Such a study would benefit from direct observation to accompany survey data collection, and possibly from an experimental design including a control group. The combination of iBuddy with other digital education activities—both related to privacy and security, or broader, like coding or media literacy activities—would also provide additional evidence.

iBuddy is just a first tentative step towards the design of simple, game-like tools designed to make users aware of how our everyday technology works. We believe that if technology providers themselves invested in this type of tools, possibly adapted both for school education and for informal settings, the long march towards a more transparent and equitable digital society would become swifter.

## Footnotes

1 In case illegal content is collected (e.g., pedo-pornographic pictures), the operator is instructed to duly report to the class teacher. In all the sessions reported in this study, this situation never happened.
